# Supplementary material for: Are carbon emissions trading and green financial instruments synergistic? -Comprehensive quantitative research based on content analysis
Source: PLoS One. 2024 Mar 7;19(3):e0298601. doi: 10.1371/journal.pone.0298601 (PMC10919601; doi:10.1371/journal.pone.0298601)
Supplement: S1 Data — (ZIP) [file pone.0298601.s001.zip › Data/地级市Z_F工作报告.zip!/╝¬┴╓/┴╔╘┤╩╨/└┤╫╘╣1⁄2╓┌║┼í╛┬φ┐╦╩2╛▌═°í┐_2017.docx]

各位代表：

　　现在，我代表市政府，向大会报告政府工作，请予审议，并请市政协各位委员提出意见。

　　一、过去四年工作回顾

　　市七届人大一次会议以来，面对错综复杂的国际国内形势和持续加大的经济下行压力，在市委的坚强领导下，在市人大、市政协和社会各界的监督支持下，市政府同全市人民一道，主动适应经济发展新常态，牢牢把握转型升级这一主线，始终坚持稳中求进，致力优化经济结构，统筹推进城乡建设，充分激发市场活力，着力保障改善民生，辽源各项事业发展呈现新局面。

　　综合实力稳步增强。与2012年相比，预计2016年地区生产总值实现800亿元，年均增长7.2%。全口径财政收入完成41.3亿元，年均增长2.06%，其中地方级财政收入完成24.2亿元，非税收入比重下降1.6%。全口径财政支出达到110.5亿元，年均增长7.6%。全社会固定资产投资完成675亿元，年均增长14.6%。实施亿元以上项目451个，竣工或投产378个。社会消费品零售总额达到226.4亿元，年均增长11.4%。外贸进出口总额达到2.89亿美元，年均增长14%。粮食生产实现“六连增”，特色农产品达到157个。规上工业总产值实现1555亿元，年均增长10.9%。服务业增加值实现275.2亿元，年均增长9.1%。民营经济占GDP比重达到58.4%，民营工业占全市工业比重超过90%，均列全省第一位。东丰县获全省县域经济综合发展考评优胜奖，东辽县项目建设现场考评位次创历史最好水平。在国务院第二次大督查中，我市作为全省唯一受表彰地区，获得国家专项建设基金、转移支付资金、划拨沉淀资金、申发企业债“直通车”和新增建设用地计划指标等奖励扶持政策。

　　转型发展步伐坚实。装备制造、农产品深加工两大主导产业占工业比重60%，高精铝加工、纺织袜业、医药健康、蛋品加工、新能源等特色优势产业及行业快速形成规模。采掘业和产能过剩行业占规上工业比重下降3.4个百分点。旅游、电商等新业态不断涌现，服务业占比提高2.3个百分点。农村土地流转适度规模经营81万亩，调减籽粒玉米种植面积5.76万亩。国家级农业标准化示范区达到11个。建立院士工作站4个、省级以上科技孵化器7个、各类研发中心96个。培育国家和省名牌产品71个、地理标志保护产品3个，驰名、著名商标达到120件。实施节能减排和污染防治项目120个，单位GDP能耗下降29.6%。我市获得27个国家级、28个省级试点示范认定。东丰县被评为全国魅力新农村十佳县和中国美丽乡村建设示范县。东辽县被列为全国现代农业示范区、吉林省率先实现农业现代化示范县。

　　城乡面貌显著变化。修编城镇化发展、土地整治、城市风貌等21个专项规划。建成辽长铁路、辽西高速，新增公路里程460公里，修建农村公路936公里。完成杨木水库输水复线、东辽河城防等重点水利工程。我市第一部地方立法《杨木水库饮用水水源保护条例》颁布实施。新建改造市政道路122条、地下管网471公里，市区集中供热能力新增672万平方米。“气化辽源”长输管线工程达到供应条件。省级园林城、生态城通过评审。建成城市公共信息平台和“智慧城市”应用项目45个，落户知名信息软件企业60户。我市被列为全国信息惠民试点城市，获得中国智慧城市示范奖。两县县城建设取得重要进展，3个乡（镇）被列为全国重点镇，辽河源镇入选全国首批特色小镇。清收林地4.1万公顷，工程造林1.8万公顷。完成100个行政村环境连片整治，10个村被评为国家和省级美丽乡村。我市被评为“最美中国生态（自然）旅游胜地、美丽乡村旅游胜地”。

　　改革开放实现突破。启动政府系统改革241项，完成125项。完成32个政府部门机构改革，建立部门权力清单和责任清单，建成政务服务、公共资源交易、农村综合产权交易“三个中心”。企业“五证合一”和个体工商户“两证整合”等商事制度改革取得显著成效，累计登记市场主体8.3万户。“营改增”、市区分税、预算公开等财税改革扎实推进，国有资产纳入规范化管理。新增政府、行业融资平台和小额贷款公司53个。农业农村改革、城镇化配套改革以及生态文明、社会体制改革取得重要进展。对外交流合作覆盖经济社会发展各个领域，成功举办国际袜交会、琵琶演奏大赛以及一系列高峰论坛，东丰农民画在联合国总部举办画展。纳入省招商引资调度系统项目达到384个，合同履约率92.7%，当期落地开工项目286个。建成3个省级出口基地，出口产品达到56种，我市被列为省级出口袜制品质量安全示范区。

　　人民生活持续改善。预计城乡常住居民人均可支配收入分别达到29129元和11302元，年均增长8.2%和7.3%。实施163件惠民实事，累计投入超过200亿元。新增城镇就业16.1万人次，转移农村劳动力71.6万人次，城镇登记失业率控制在3.4%以内。企业养老金、失业金标准分别提高47%和134%，城乡低保分别提标46.5%和65%。调整机关事业单位工资、提高公积金缴存比例、发放乡镇工作人员补贴等政策得到落实。建立健全社会救助政策，惠及8万余户困难家庭。城居保参保率、新农合参合率分别达到96%和99.4%，政策报销比例75%，基本药价下降30%。在全省率先启动城镇居民大病医疗保险，受益群众达到10939人。已完成棚户区全部改造任务的90%，惠及居民9.48万户。改造“暖房子”712栋，整治老旧小区83个。城市公交公营比例达到62.4%。市区燃气普及率达到97%。24小时供水能力达到97%。7.69万农村居民饮水安全问题得到解决。

　　社会事业全面进步。完成薄弱校改造项目87个，4个县（区）全部通过国家义务教育基本均衡考核验收。我市列入全国首批现代学徒制试点和首批老工业基地产业转型技术技能人才双元培育改革试点，被评为第二批全国高校实践育人创新创业基地。社会事业领域投资最大的职教园区启动建设。建立分级诊疗、基本药物等制度，建设标准化村级卫生室488个，基本医疗服务体系覆盖城乡。二战高级战俘营旧址展览馆、矿工墓陈列馆、矿山博物馆等文化设施投入使用，龙首山古城及相关遗迹挖掘保护开发取得重大进展。成功承办省第十七届运动会。广播电视完成高清技术装备改造，实现数字化播出。城乡养老服务机构达到89个，“三维养老”模式初步形成。扶持残疾人创业就业2700人，开展康复医疗2.4万人。民族团结、宗教和顺局面持续巩固。国防动员、双拥共建和军民融合发展基础更加牢固。我市被评为全国人民防空先进城市。外事侨务、方志、档案、气象、地震等工作和妇女、儿童、老龄、关心下一代等各项事业协调向前推进。

　　法治建设富有成效。贯彻依法治市总体要求，坚持系统治理、综合治理、源头治理相结合，建立完善重大行政决策程序和合法性审查、政府法律顾问等一系列制度机制，办事依法、遇事找法、严格规范公正文明执法环境进一步形成。法治宣传教育不断强化，我市荣获“六五”普法全国先进城市。建成立体化安防体系，促进平安辽源建设，刑事、治安案件分别下降17.4%和11.2%，群众安全感满意度保持全省领先，连续5年获得全国见义勇为城市奖。注重依靠法治手段调处社会矛盾，及时妥善化解信访突出问题。我市被评为全国创新社会治理优秀城市。坚持严格监管与专项整治同步推进，连续7年获得省政府安全生产目标责任制考核一等奖，连续32年无重大森林火灾，食品药品安全管理成效显著。

　　行政效能不断提升。扎实开展党的群众路线教育实践活动、“三严三实”专题教育和“两学一做”学习教育，整改21个突出问题，建章立制24项。认真贯彻中央“八项规定”精神，政府会议文件减少20%，市级“三公”经费下降10.4%。精简市级行政审批事项75%，审批时效提高55%，非行政许可事项实现“零审批”。自觉接受人大的法律监督、工作监督和政协的民主监督，办理代表建议393件，政协提案465件。深入推进政务公开，畅通“五位一体”诉求渠道，全省首家政府公共服务热线平台建成运行。切实强化党风廉政建设“两个责任”“一岗双责”，公共资金、重大工程、国有资产资源处置审计监督实现全覆盖，严肃查处了一批政府公职人员违法违纪问题。

　　各位代表，刚刚过去的2016年，市政府全面贯彻市委决策部署和市“两会”确定的目标任务，重点抓了六个方面工作，实现了“十三五”良好开局。一是保持经济稳健运行。分行业按企业落实精准调控政策，利源精制、麦达斯铝业、东北袜业、厚德食品、鸿图锂电、巨峰生化等骨干企业产销两旺。推动百康、迪康等医药企业分别与重庆三圣、武汉九州通实现整合重组。规上工业企业新增15户。50个投达产技改项目贡献规上工业产值增量的80%。全市工业用电量增幅由负转正，比上年增长10%。粮食产量达到47亿斤，比上年增长5%。旅游业总收入增长25%，电商线上交易额达到15亿元，中农农副产品批发市场、凯旋王国水上乐园、御峰城市广场、新世界彩悦城相继投入运营，全市三产对经济增长的贡献率达到30%。二是强化项目建设和招商引资。实施5000万元以上项目207个，建成112个。积极对接国家支持新一轮东北振兴政策，谋划储备转型项目200个。东北袜业产业链改造升级项目收益债作为全国首个民营企业债获准发行。精准对接招商项目122个，落地建设36个。积极参与省组织的重大招商活动，签约项目22个。引进域外资金570亿元，实际利用外资3亿美元。三是实施供给侧结构性改革。积极推动鑫达钢铁复产合作和金刚水泥错峰生产，支持辽矿集团职工家属住宅区分离移交“三供一业”职能，引导矿山装备、建筑机械行业转产新产品。加大商品房回购力度，保障房货币化安置比例超过50%。优化存量政府债务结构，筹建城投控股集团，落实置换债券16.4亿元。千方百计降低企业生产要素和制度性交易成本，通过推行“营改增”等措施为企业减税2.9亿元。启动实施系列补短板攻坚行动。四是进一步改善营商环境。全面开启重点项目审批绿色通道，审批环节减少11.8%，即办件占比92.5%。我市在全省“放管服”改革满意度测评中位列第一名，民间投资比上年增长16.2%。新增小微企业400户、个体工商户4900户。科技企业孵化器在孵企业205户，聚集创客2100余人。东北袜业实施“淘宝5000”计划，电商创业培训2000余人。五是启动城市规划建设管理提挡升级攻坚。各项建设工程和专项整治基本完成年度计划任务。开工建设伊开高速辽源段二期，启动集双高速辽源段征地拆迁和“引松入辽”辽源干线工程，积极推进辽源机场建设前期工作。杨木水库水源地保护取得突破性进展。长输管道天然气用户安装1.15万户。改造人民大街等59条街路，敷设各类地下管线74.7公里。南部新城地下综合管廊建成8.2公里。完成林苑公园升级改造一期工程，新建改造城市绿地70块、景观带18条。集中治理“五乱”收到实效，纠治各类违法问题5.6万件，拆除违建6.3万平方米。六是推进精准扶贫、精准脱贫。落实“六个一批”精准扶贫方略，创新“五点双享两救助”保障机制，坚持五个精准发力，组织实施各类扶贫项目275个，累计投入3.23亿元，实现农村建档立卡贫困人口稳定脱贫1万人。

　　各位代表，过去几年，我们在应对各种困难和挑战中奋勇前行，取得的成果来之不易。这是市委正确领导的结果，是市人大、市政协和各民主党派、工商联、人民团体、无党派人士鼎力支持的结果，是全市各县（区）、各部门、各企业和广大干部群众团结拼搏的结果，是老领导、老同志和驻辽部队、中省直单位及海内外朋友热情相助的结果。全市人民万众一心、和衷共济，弘扬了中国特色社会主义和中国梦的时代主旋律，凝聚了辽源转型发展的强大正能量。在这里，我代表市政府向大家表示衷心的感谢和崇高的敬意！

　　总结成绩的同时，我们也清醒地看到存在的问题和工作的差距。主要是：我市经济总量不大，产业总体实力不强，抗风险能力较弱，供需两侧的结构性矛盾也很突出。体制机制创新任务仍然艰巨繁重，科技、人才、资金等方面存在短板。政府债务负担重，持续增收难度较大，制约了城市建设和民生投入。社会治理的科学化、法治化水平有待提高，绿色发展、安全发展的基础还不够牢固。部分公职人员的思想观念、工作作风和服务效率与转型攻坚的要求不相适应。我们一定增强责任意识、忧患意识、担当意识，下更大气力解决这些问题，竭尽全力做好政府工作，决不辜负全市人民的期望和重托。

　　二、今后五年目标任务

　　未来一个时期，宏观经济下行压力不会根本缓解，区域竞争日趋激烈。面对新旧矛盾叠加、风险隐患交织的挑战，我们必须做好爬坡过坎、滚石上山的准备。同时，更要看到，国家支持新一轮东北振兴的措施陆续出台，我省全面振兴发展正处于优势加速释放、动能加速转换、活力不断增强的重要战略机遇期，辽源业已形成接续替代产业体系框架，并且拥有良好的社会政治生态。只要我们保持定力，持续发力，抢抓用好各种政策机遇，积聚释放各种积极因素，辽源这座充满潜能的城市一定能够行稳致远、愈加绽放活力，不断迎来更加美好的明天。对此，我们充满信心。

　　今后五年，我们要深入贯彻市七次党代会精神，牢固树立和实践新发展理念，坚持稳中求进工作总基调，以推进供给侧结构性改革为主线，着力培育优势产业，调整经济结构，完善体制机制，鼓励创新创业，保障改善民生，统筹推进经济、政治、文化、社会、生态文明建设，确保如期全面建成小康社会，进一步开创辽源创新转型发展新局面。

　　经济社会发展的主要预期目标是：到2021年，地区生产总值年均增长7%左右，第三产业占比达到40%左右。民营经济占比达到60%以上。城乡常住居民人均可支配收入年均分别增长7%和9%左右。居民消费价格指数控制在4%以内。城镇登记失业率控制在3.9%以内。城镇化率达到58.5%以上。单位GDP能耗和主要污染物减排完成省下达计划。落实中突出六项任务：

　　一是以打造东北地区乃至全国资源型城市转型示范市为目标，着力构筑产业新优势。更加注重从供给侧入手，厚植产业根基，突出发展实体经济，努力走出一条质量更高、效益更好、结构更优、优势充分释放的转型新路。积极对接“中国制造2025”、“互联网+”行动及国家产业振兴政策，谋划推进一批有需求潜力、能弥补发展短板、具有创新优势和支撑作用的重大项目。把培育壮大主导和特色优势产业作为重中之重，促进一二三产衔接和“新四化”融合，加快推进区域经济创新转型升级。全力推动经济开发区和工业集中区扩容提质升级，打造高精铝加工、纺织袜业、医药健康、蛋品加工、新能源等百亿级产业集群。建设吉林省农业精品市和畜牧业强市。推动服务业速度规模和质量效益双提升。

　　二是以创建国家可持续发展实验区为载体，着力培育发展新动能。着眼于增强发展的内在活力和内生动力，集成各类创新资源，做好有中生新、无中生有两篇文章，推动要素驱动向创新驱动转变。积极参与关键共性技术协同攻关，加强研发、中试、转化、推广、应用等环节精准对接，抢占产业发展制高点。用先进适用技术改造提升传统产业，培育装备制造、农产品深加工、高精铝加工等8个产业创新链，努力将辽源经济开发区建设成为国家高新技术产业集聚区。把品牌建设作为培育新动能的重要载体，以打造品牌提升产品附加值和产业竞争力。统筹推进国家可持续发展实验区和相关试点、示范建设，放大政策的“共振”效应，在经济发展的同时实现资源有序利用和生态环境质量改善。到2021年，全市研发投入占GDP比重达到1.5%以上，科技进步贡献率提高到60%。

　　三是以建设东北地区民营经济发展改革示范城市为契机，着力完善“双创”新机制。实施市场准入负面清单管理，引导民营资本进入特许经营和社会服务领域，推进政府和社会资本合作。深化民营经济配套改革，形成政府激励创业、社会支持创业、劳动者勇于创业机制，发展一批主业突出、核心竞争力强的民营企业集团。以南部新城职教园区为依托，建设中德创新中心、“双创”服务平台、“双创”孵化示范基地。着力创造振兴实体经济的政策环境和创新环境，在加强有利于民营经济可持续发展的人才队伍建设上实现突破。完善市场监管体系，强化纳税人意识，提升政府服务水平，致力构建公平的市场秩序、“亲清”的政商关系和良好的营商环境。

　　四是以争创全国生态修复、城市修补试点为基础，着力展现城市新风貌。严格实施主体功能区划，明确生态保护红线，建立空间治理体系，打好大气、水、土壤污染防治三个战役，基本消除重污染天气和东辽河干流出境断面劣五类水体。有计划、有步骤地修复被破坏的山体、湿地、植被，积极推进采煤沉陷区、采矿废弃地修复和再利用，深入开展封山育林、林地清收还林，加快构筑起吉林省中南部绿色生态屏障。有序实施城市修补和有机更新改造，下力气提升城市建设管理水平，使城市形态与山水环境相和谐，城市功能与城市形象相统一，城市内在品质与外在风貌相协调。要通过我们实实在在的努力，让青山依旧、碧水畅流、蓝天常驻，百姓神清气爽，城市生机盎然。

　　五是以融入吉林省向南开放战略为突破口，着力形成开放新格局。树立开放性思维，在“一带一路”和长吉图开发开放、全省中部创新转型核心区建设布局中找准定位。大力推动铁路、公路、机场等重大工程，打开以辽源为节点的吉林省向南开放新通道。积极寻求与浙江省等发达地区的产业契合点、与周边地区的利益共同点，主动开展对口合作。加强辽河流域城市联合协作，在四平、辽源、铁岭、通辽经济协作区建设中发挥重要作用。扩大与东北亚地区和有关国际组织成员国之间的经贸交流，充分挖掘政府、企业两类资源，充分利用国际、国内两大市场，充分调动供需双方两个积极性，努力促成和落地一批重大合作项目，推动更深层次、更高水平对外开放。

　　六是以增进全市人民福祉为落脚点，着力创造百姓新生活。落实以人民为中心的发展理念，想群众所想、急群众所急、解群众所困，确保如期全面建成小康社会。持续增加基本公共服务和公共产品供给，不断提高城乡居民收入水平和生活品质，努力在学有所教、劳有所得、病有所医、老有所养、住有所居上持续取得进展。坚决打好打赢扶贫开发攻坚战，提高贫困群众自我发展能力。弘扬社会主义核心价值观，提高公民文明道德素质，丰富群众精神文化生活，凝聚传递社会正能量。统筹推进法治辽源、平安辽源、诚信辽源建设，织紧织密公共安全网，建设辽源人民安居乐业的和谐家园。

　　三、2017年工作安排

　　按照市七次党代会总体部署，综合研判当前形势和各方面条件因素，确定今年经济社会发展的主要预期目标是：地区生产总值增长7%左右，地方级财政收入增长3%，全社会固定资产投资增长8%左右，社会消费品零售总额增长8%左右，外贸进出口总额增长5%以上。城乡常住居民人均可支配收入与经济增长基本同步。城镇登记失业率控制在3.9%以内。节能减排必保完成省计划。为完成上述目标，我们将统筹协调，重点抓好以下八个方面工作：

　　一是努力保持经济稳定增长。发挥有效投资关键作用。实施5000万元以上项目125个，其中10亿元以上项目20个。滚动谋划和储备重大项目200个。重点推动麦达斯铝业2.35万吨挤压生产线、鸿图锂电三期、中聚新能源二期、聚源重型石墨深加工和百康药业、莎普爱思强身药业扩能改造等产业类项目，集双高速、城市地下综合管廊等基础设施建设项目，中德东辽河源头现代生态农业示范区等农业项目，中农农副产品批发市场二期等服务业项目。突出工业经济主导地位。规上工业总产值、增加值分别增长8%和8.5%，规上企业发展到320户，扩能技改项目投达产34个。全面提高经济运行质量和要素配置效率，强化对企业脱困转型、创新发展的财政、税收等政策支持。引导金融机构加大支持力度，放大“助保池”融资功能，鼓励开展股权融资、差异化信贷，着力解决中小企业融资难问题。推进“三去一降一补”。继续控制钢铁、煤炭、水泥行业新增产能，淘汰落后产能，优化存量产能，加大处置“僵尸企业”力度。积极推动商业房地产去库存，消化和转化粮食库存。着力化解地方债务和金融风险，降低企业杠杆率。积极探索低费率担保、直购电试点、企业稳岗补贴、物流对接互补等降本增效措施。深入实施补短板系列攻坚行动。

　　二是加快升级现代产业体系。全面提振实体经济。壮大高精铝深加工产业，推动骨干企业扩能改造，保持产业领先优势。完善东北袜业“平台+服务”模式，建设全国棉袜产业知名品牌示范区。以厚德食品为标杆，引导农产品深加工企业向精深加工和全产业链方向发展。支持医药企业资源整合和资本重组，加快建设吉林省化药基地。引导锂电隔膜、锂电池、碳纤维材料等战略性新兴产业规模化、集群化发展。全力支持辽矿集团等重点企业转型升级，积极帮助矿山装备、建筑机械等传统行业转产新产品、开拓新市场。加快推进城区老工业区搬迁改造。大力培育现代服务业。加强特色资源产品的综合开发，用足用活辽源的文化元素，实施鴜鹭湖生态旅游度假区、凯旋王国创意园、扎兰芬围民俗园、房车营地、国家级登山步道等20个旅游项目。打造辽河源、金州等生态旅游特色小镇。支持东北袜业实施“淘宝5000”计划，引导线上线下融合发展，做实做强阿里巴巴辽源产业带。发扬“工匠精神”，强化质量意识，打造一批知名品牌和“百年老店”。积极培育健康养老、文化创意、科技服务等新业态，推动服务业向专业化和价值链高端延伸。着力推进农业供给侧结构性改革。继续调整种植业结构，增加订单生产和产加销衔接，探索土地稳定流转和农民稳定增收保障机制。完善农田水利基础设施，新增20万亩高标准农田，实施20个农业科技项目。完成吉东种业改制重组。支持东丰县建设全省三产融合发展示范县和国家梅花鹿产业核心区。扎实推进农村土地“三权分置”，支持东辽县建设全国农村土地经营权抵押贷款试点示范县。壮大家庭农场、专业大户、农民合作社等新型经营主体，在建设现代农业产业集聚区方面迈出新步伐。

　　三是积极培育发展新动能。加快科技创新步伐。加强与科研院所和高校合作，促进生物医药、电子信息、装备制造、新能源、新材料等领域科技成果转化。加快移动互联网、物联网、云计算、大数据等新一代信息技术应用，提升制造业智能化层级。推进医药、铝合金深加工高新技术产业基地和产业技术创新联盟及中试中心建设。建成国家袜制品及原材料质量监督检验中心。辽源经济开发区申报省级高新技术产业集聚区。促进大众创业、万众创新。鼓励科研人员在职创业、离职创业，建成9个市级以上大学生创业园。鼓励农村青年带头创办家庭农场、领办农民专业合作社和兴办农业社会化服务组织，建成市级以上农民返乡创业基地20个。实施新兴产业“双创”行动计划，科技企业孵化器聚集创客3000人以上。新增小微企业400户以上。大力实施人才战略，推进招才引智和实用型人才培育，全面开展老工业基地产业转型技术技能人才双元培育改革试点，加快全国高校实践育人创新创业基地建设。推进体制机制创新。深化“放管服”改革，优化发展软环境，推行“互联网+政务服务”，完成“一号一窗一网”建设任务。健全重大项目领办、代办和网上并联审批制度，加强事中事后监管“双随机、一公开”。深入推进商事制度改革，推动信息共享监管和社会共治协同。创新投融资体制，启动运营城投控股集团，建立向全社会公开的PPP项目库。支持民营企业股份制改革和登陆资本市场。创新信贷产品，推广创业担保贷款，支持农村电商“双创”。稳步推进农民住房财产权抵押贷款试点。

　　四是全方位推动开放融合。加快融入区域经济圈。借助既有通道，密切与长吉、长平、哈长城市群、环渤海经济圈之间的联系。推动以辽源为节点的长春、沈阳产业走廊联通，开展汽贸、轨道交通等领域协作配套。建设与通化、西丰联接的健康产业走廊，加强医药、梅花鹿产业合作。争取纳入吉林旅游大环线，融入长白山生态旅游圈。积极寻求融入“一带一路”和我省向南开放战略新的切入点，拓展与有关国家和地区在现代农业、装备制造等领域的互利合作。制定实施与四平、铁岭、通辽等城市的经济协作计划。启动辽源内陆干港建设准备工作。开展务实精准招商。以我市主导产业和特色优势产业为主攻方向，积极承接长三角、珠三角和长江经济带产业转移，推进经贸洽谈和投资项目落地。以本地骨干企业为核心，吸引上下游关联企业，合作建设特色产业集群。全年引进域外资金660亿元，实际利用外资3.5亿美元。不断提高出口产品质量和市场份额，申报国家级出口袜制品质量安全示范区。

　　五是统筹推进新型城镇化。加快建设重大基础设施。续建伊开高速辽源段二期，启动集双高速辽源段建设，新建农村公路100公里，改造中德东辽河源头现代生态农业示范区主干路。积极推动辽源机场、四通客运专线和辽开、四梅铁路电气化改造列入实施计划。加快建设“引松入辽”辽源干线、东辽河城区段防洪、中小河流治理等水利工程，启动建设配套净水厂和污水处理厂。加快“气化辽源”入户，新增长输管道天然气用户2万户。扎实推动南部新城建设，力争职教园区一期达到入驻条件。全面推进城市规划建设管理提挡升级攻坚。实施市政“五路”改造工程，整修巷路和人行步道30条。改造各类管网50公里，建设地下综合管廊4公里。新建改造5条景观带、20块街头绿地，完成林苑公园二期工程。有序推进海绵城市建设。实施东辽河城区段景观带和人民大街等街路亮化工程。大力规范城市交通秩序，创建市容市貌专项整治3条示范街、8条严管街。实施“智慧城市”大数据基础平台和31个智慧社区建设，推动信息及各类资源共享。统筹加强县城和重点镇建设。完善基础设施配套，提升两县城市功能，增加人口、要素集聚能力，扶持发展一批工业主导型、生态旅游型、历史文化型、商贸流通型特色镇，推进县域经济升级晋位。积极推动户籍制度改革和居住证制度双落地，加快基本公共服务常住人口全覆盖。灵活运用财政贴息、奖补政策，引导带动民间资本、域外资金投入新型城镇化建设。同步推动新农村建设。

　　六是全力保障改善民生。继续推进脱贫攻坚。坚持精准脱贫与整村推进相结合，建立健全产业到户到人的扶持机制，发展农林、旅游、电商等特色产业。加大财政、金融、土地等政策扶持力度，创新政府、企业和社会组织联动机制，强化医疗、教育、养老、助残等专项扶贫，确保1万贫困人口实现脱贫。不断完善公共服务。城镇新增就业3.3万人，新增创业3000人，失业人员再就业6000人，转移农村劳动力15万人次以上。继续提高养老、失业金发放标准，完善各项救助制度。推动城乡义务教育一体化改革发展，落实特殊群体助学政策，确保进城务工人员随迁子女平等接受义务教育。实施全民健康计划，启动健康卫生“七大工程”。建立多层次养老服务体系，新增3个医养融合养老机构，增加床位800张。解决一批群众关注的热点问题。开工建设保障房6400套，回迁居民3500户。有计划地实施城市危楼改造，继续推进“暖房子”工程。建立物业企业与业主双向约束机制，在10个小区开展试点。新建一批立体式停车场，延伸部分公交线路，合理设置公交站点。实现市区重点公共场所无线网络免费覆盖。实施市区小学营养配餐计划。启动南部新城全民健身中心建设，增加一批公共文体设施。

　　七是有效开展生态文明建设。加强水污染防治。推进杨木水库人工湿地建设，启动水源地保护“三网同筑”工程。推行“河长制”，综合整治域内河流流域环境和仙人河黑臭水体。加强大气污染防治。强化工业废气、机动车尾气、建筑扬尘等动态监控，取缔建成区10蒸吨以下燃煤锅炉，加快普及使用天然气等清洁能源。加强土壤污染防治。科学合理划定畜禽禁养区，有效控制农业面源污染。大力推进矿山环境治理，继续开展规划区内居民和工企户搬迁，依法解决私搭乱建问题。加强生态修复保护。工程造林1300公顷，绿化村屯70个，道路、河岸绿化162公里。加快推进矿山湿地公园、寒葱顶森林公园、乌龙山自然保护区建设。启动生态修复、城市修补试点申报工作。积极发展循环经济。推行垃圾分类制度，加快天楹垃圾焚烧发电厂达产达效。推进低效用地二次开发。探索农村秸秆禁烧和综合转化利用有效途径。力争省级循环经济试点市建设取得较大突破。

　　八是切实强化社会治理。深入开展“七五”普法，加强以学习和宣传宪法为核心的法治宣传教育。深入推进平安辽源建设，强化“十大领域”风险防控，完善立体化安防体系，保持对违法犯罪的严打高压态势。健全社会矛盾纠纷化解机制，确保群众诉求渠道畅通、合理诉求得到妥善解决。落实安全生产“党政同责、一岗双责、齐抓共管、失职追责”责任体系，及时排查和消除安全隐患，坚决遏制较大以上生产安全事故。强化食品药品动态监管，逐步推广农产品质量安全生产地准出、市场准入和信息可追溯制度。加强应急预警机制建设，健全防灾减灾救灾体系。构建社会综合服务平台和社会信息服务网络，促进政府行政管理和社会自我管理有效对接。加强社会诚信建设，让诚信者权益得到充分保障，让失信者付出相应代价。加强全民国防教育、国防动员双拥共建，强化驻辽部队和国防动员系统改革服务保障，推进军民融合深度发展。

　　各位代表，毫不懈怠地抓好自身建设，始终是我们完成各项任务的坚实保障。要适应新形势，把握新要求，增强政治意识、大局意识、核心意识、看齐意识，驰而不息改进作风，不断提高施政能力和水平，努力建设人民满意的服务型政府。

　　我们将尊崇法治，依法行政。认真贯彻法治政府建设实施纲要，健全重大行政决策程序，严格履行各项法定职责，把政府工作全面纳入法治轨道。落实行政执法责任制，推进城市综合执法改革，依法解决征收拆迁、城市管理、社会治理等方面的难点问题。政府公职人员要心中高悬法律明镜，手中紧握法律戒尺，切实把对法治的尊崇、对法律的敬畏转化为思维方式和行为方式。任何人都不得违法越权、任性妄为。

　　我们将扎实根基，俯首为民。坚持民之所望政之所向，虚心向群众问计求教，广泛听取和及时回应群众的呼声诉求。坚持以增进百姓福祉为指南，在公共资源配置、市政工程安排、财政资金使用上优先向民生倾斜，多做雪中送炭的工作，满腔热情为群众办实事。要自觉接受市人大及其常委会的法律监督、工作监督和市政协的民主监督及社会舆论监督，让各界群众参与政府决策、检查执行情况、评价落实结果，使政府工作更接地气、得民心，充分体现人民的意愿。

　　我们将扛起责任，勇于担当。树立功成不必在我的理念，坚持一张蓝图绘到底，扎实做好打基础、利长远的工作，决不急功近利搞“形象工程”和“政绩工程”。增强克难攻坚的胆识，集中力量抓大事、破难题、啃硬骨头，看准的事情坚定干、抓到底，决不对份内的职责推诿躲闪。要把政治责任转化为推动发展、改善民生的履职动力，做到日常工作能尽责，难题面前敢负责，出现过失勇担责，真正把使命和责任扛在肩头。

　　我们将创新管理，从严治政。始终保持昂扬向上的精神状态和朴素务实的工作作风，狠抓本领提升和工作落实，提高政府执行力和公信力，确保政令畅通。探索“督考合一”办法，健全督查激励问责机制和容错纠错机制，鼓励干事创新创业，强化绩效管理的导向作用。加强行政监察、审计，持之以恒纠正“四风”，坚决整治为官不为、懒政怠政行为，严肃查处寻租贪腐、失职渎职问题，切实做到干部清正、政府清廉。

　　各位代表，全面建成小康社会、加快创新转型发展，是全市人民的殷切期盼，是我们义不容辞的庄严使命。让我们紧密团结在以习近平同志为核心的党中央周围，在省委、省政府和市委的坚强领导下，凝心聚力，开拓奋进，真抓实干，为不断谱写辽源人民美好生活新篇章而努力奋斗!
